# V_2_O_5_-Assisted Low-Temperature Sintering and Microwave Dielectric Properties of (1 − x)Li_2.08_TiO_3_–xLi_2_ZnTi_3_O_8_ (x = 0.3−0.7) Ceramics for LTCC Applications

**DOI:** 10.3390/ma19010094

**Published:** 2025-12-26

**Authors:** Yu-Seon Lee, Kyoung-Ho Lee

**Affiliations:** Department of Electronic Materials, Devices, and Equipment Engineering, Soonchunhyang University, 22, Soonchunhyang-ro, Asan-si 31538, Chungnam, Republic of Korea; ys1ee@sch.ac.kr

**Keywords:** Li_2.08_TiO_3_‒Li_2_ZnTi_3_O_8_ composites, V_2_O_5_ sintering aid, low-temperature densification, liquid-phase sintering, high *Q* × *f*, near-zero *τ_f_*, microwave–dielectric properties, Ag co-firing compatibility, LTCC

## Abstract

A new composite microwave–dielectric system, (1 − x)Li_2.08_TiO_3_-xLi_2_ZnTi_3_O_8_ (x = 0.3–0.7), was systematically investigated to identify the optimal composition for low-temperature co-fired ceramic (LTCC) applications by correlating sintering behavior, microstructural evolution, and microwave–dielectric properties. Although the undoped compositions exhibited excellent intrinsic dielectric performance, they required sintering at 1100 °C, making them incompatible with Ag-based LTCC processing. Among the investigated formulations, 0.6Li_2.08_TiO_3_–0.4Li_2_ZnTi_3_O_8_ was identified as the most suitable base composition. To reduce the sintering temperature, 0.3–1.0 wt.% V_2_O_5_ was introduced as a sintering aid, enabling densification at 900 °C for 30 min (97.0% relative density) while preserving the coexistence of Li_2.08_TiO_3_ and Li_2_ZnTi_3_O_8_ without XRD-detectable secondary phases. Microstructural observations indicated that V_2_O_5_ promoted liquid-phase sintering, leading to enhanced densification and Li_2.08_TiO_3_-selective abnormal grain coarsening without altering the intrinsic permittivity. Complementary dilatometry provided process-level evidence for this liquid-phase sintering mechanism: large total shrinkage at 900 °C ($∆L/L_{o}$≈ −17–19%), earlier $T_{onset}$/$T_{peak}$ with $T_{peak}$ lowered by ~250 °C, and an increased $R_{peak}$, collectively supporting 900 °C/30 min as the practical firing window. The optimized 0.6Li_2.08_TiO_3_–0.4Li_2_ZnTi_3_O_8_ composition containing 0.3 wt.% V_2_O_5_ exhibits excellent microwave–dielectric properties ($\varepsilon_{r}$ = 23.32, *Q × f* = 68,400 GHz, and $\tau_{f}$ = −1.55 ppm/°C). Higher V_2_O_5_ contents (>0.3 wt.%) caused a gradual reduction in *Q × f* due to increasing microstructural non-uniformity. Ag co-firing tests confirmed electrode stability with no interfacial reactions at 900 °C for 30 min. Overall, 0.3 wt.% V_2_O_5_-assisted 0.6Li_2.08_TiO_3_–0.4Li_2_ZnTi_3_O_8_ provides a practical sub-950 °C processing window that satisfies key LTCC requirements, including moderate permittivity, high *Q × f*, near-zero $\tau_{f}$, and compatibility with Ag electrodes.

## 1. Introduction

Microwave–dielectric materials have become essential components in modern information and communication technologies, including in the Internet of Things, Intelligent Transportation Systems, and 5G microwave communication systems [1,2,3,4]. The rapid expansion of these technologies has driven growing demand for high-performance microwave–dielectric materials and continued device miniaturization. In this context, low-temperature co-fired ceramics (LTCCs) have emerged as a key enabling technology. LTCCs enable the miniaturization of communication components, integration of passive elements, and reduction in manufacturing costs. LTCCs have also become central to a broad range of applications, including electronic packaging and microwave system research [5,6,7,8,9,10,11,12].

To develop LTCC dielectric materials for 5G microwave applications, several key requirements must be met: (1) a low sintering temperature (≤950 °C) to enable co-firing with highly conductive Ag electrodes, (2) a high quality factor (*Q* × *f* > 50,000 GHz) to maintain strong frequency selectivity, (3) a near-zero temperature coefficient of resonant frequency ($\tau_{f}$) to ensure thermal stability, (4) minimal chemical reactivity with Ag electrodes, and (5) a moderate-to-high relative permittivity ($\varepsilon_{r}$ ≥ 20), as higher permittivity reduces the effective guided wavelength ($\lambda_{g}$ ≈ $\lambda_{0}$/$\sqrt{\varepsilon_{r}}$), facilitating device miniaturization and increasing capacitance density in multilayer LTCC structures. When combined with a high *Q* × *f*, this permittivity requirement enables compact, low-loss microwave components for 5G applications [10,11,12]. Discovering compositions that meet all these criteria is critical for next-generation microwave wireless systems, from 5G to 6G.

Among various design strategies, achieving a near-zero $\tau_{f}$ remains particularly challenging, as it must be balanced with other key dielectric performance metrics. A widely adopted and effective approach is the design of composite dielectrics that combine a positive-$\tau_{f}$ phase with a negative-$\tau_{f}$ phase, allowing mutual compensation and maintaining overall phase stability [13,14,15].

Based on this framework, we focused on the Li_2_TiO_3_–Li_2_ZnTi_3_O_8_ pair as a chemically compatible composite platform for $\tau_{f}$ compensation. In this system, Li_2.08_TiO_3_—a Li-enriched derivative of Li_2_TiO_3_—serves as an enhanced positive-$\tau_{f}$ end member, exhibiting superior dielectric performance ($\varepsilon_{r}$ = 24.6, *Q × f* = 66,000 GHz, $\tau_{f}$ = +22.1 ppm/°C) and a lower sintering temperature (1100 °C) compared with conventional stoichiometric Li_2_TiO_3_ [16]. Therefore, Li_2.08_TiO_3_ was selected instead of Li_2_TiO_3_ as the positive-$\tau_{f}$ end member in this study. In contrast, Li_2_ZnTi_3_O_8_, a negative-$\tau_{f}$ dielectric with a stable spinel structure, has been widely studied for its favorable properties ($\varepsilon_{r}$ = 25.6, *Q × f* = 72,000 GHz, $\tau_{f}$ = −11.22 ppm/°C) [17,18,19,20]. For brevity, Li_2.08_TiO_3_ and Li_2_ZnTi_3_O_8_ are hereafter denoted LT and LZT, respectively, and composites are written as (1 − x)LT–xLZT. Motivated by the strong chemical stability reported for LT–LZT composites, we synthesized a composite comprising positive-$\tau_{f}$ LT and the negative-$\tau_{f}$ LZT, achieving a material with high *Q × f*, excellent chemical stability, and tunable near-zero $\tau_{f}$. However, this composite requires a sintering temperature of 1100 °C, exceeding the LTCC processing limit (≤950 °C) and posing a significant barrier to practical implementation.

To address this limitation, we introduced V_2_O_5_ (melting point ≈ 690 °C) as a sintering aid. V_2_O_5_ is a low-melting oxide that promotes liquid-phase sintering and acts as a reactive flux, significantly lowering the sintering temperature while preserving the dielectric performance of the base composite when used in optimized amounts [21,22,23,24,25,26,27,28]. This additive was selected as a candidate flux because prior studies have shown that trace amounts of V_2_O_5_ can activate liquid-phase-assisted densification in various Ti-based oxide systems, suggesting that it may also be effective in the LT–LZT system [21,22,24,28]. Using this additive, we systematically investigated the effect of V_2_O_5_ content on sintering behavior and microwave–dielectric properties. Our ultimate objective is to demonstrate the practical applicability of the developed composition as an LTCC dielectric that maintains a high *Q × f*, provides thermal stability, and exhibits reliable co-firing compatibility with Ag electrodes. Compared with the Li_2_O–B_2_O_3_–SiO_2_–CaO–Al_2_O_3_ glass approach reported by Lei et al. [15], our glass-free V_2_O_5_-flux route enables co-firing at 900 °C for 30 min, ensures Ag compatibility, and achieves near-intrinsic $\varepsilon_{r}$, high *Q × f*, and near-zero $\tau_{f}$. This approach provides a practical and effective pathway for LTCC integration.

## 2. Materials and Methods

High-purity Li_2_CO_3_, ZnO, and TiO_2_ powders (≥99.9%, Kojundo Chemical Laboratory, Sakado, Japan) were used as the starting materials. Li_2.08_TiO_3_ and Li_2_ZnTi_3_O_8_ powders were synthesized by calcining stoichiometric mixtures of Li_2_CO_3_–TiO_2_ and Li_2_CO_3_–ZnO–TiO_2_, at 850 °C for 4 h and at 900 °C for 4 h, respectively.

The composites were designed within the compositional range of (1 − x)LT–xLZT (x = 0.3–0.7) where x represents the volume fraction of the LZT phase in the composite. For wet milling, ethanol (≥97.5%, Daejung Chemicals, Siheung-si, Republic of Korea) was used as the solvent, and the mixture was milled for 24 h with zirconia balls. The milled powder was then mixed with 1 wt.% polyvinyl alcohol (PVA) as a binder using a three-roll mill (EXAKT 50I, EXAKT, Norderstedt, Germany) to form granules, which were dried at 90 °C and sieved through a 60-mesh screen. For V_2_O_5_-doped samples, 0.3, 0.5, 0.7, and 1 wt.% V_2_O_5_ were added to the 0.6LT–0.4LZT composition, and the composites were prepared following the same procedure. For the V_2_O_5_-doped mixtures, SEM examination of the wet-milled LT–LZT powders confirmed that the particles remained uniformly dispersed without pronounced agglomeration, with particle sizes in the submicron range (600–700 nm).

To analyze the sintering behavior and measure the $\varepsilon_{r}$, *Q × f*, and $\tau_{f}$ values of the (1 − x)LT–xLZT (x = 0.3–0.7) composites, the granulated powders were uniaxially pressed into cylindrical pellets (8 mm in diameter and 4 mm in thickness) at 150 MPa. The pressed density was controlled at ≈59% across compositions. The pellets were preheated at 500 °C for 10 h to remove the PVA binder, followed by sintering at 1100 °C for 2 h. For the V_2_O_5_-doped samples, the same pressing and preheating conditions were applied, and sintering was performed at 650–1100 °C for 0.5–6 h. For most compositions, sintered density and microwave–dielectric properties ($\varepsilon_{r}$, *Q × f*, and $\tau_{f}$) were reported as the mean values of five specimens. However, for the V_2_O_5_-free 0.6LT–0.4LZT compositions sintered at 900 °C for 30 min, only a single pellet could be measured because the relative density was as low as 61%. Therefore, no error bar (standard deviation) is provided for this data point.

The bulk densities ($\rho_{\mathrm{bulk}}$) of the sintered specimens were measured by the Archimedes method, and the relative densities were calculated from the measured bulk densities and the theoretical densities ($\rho_{t}$). The theoretical density of the composites ($\rho_{t}$) was calculated using Equation (1):

(1) $$\rho_{t}\;=\;\frac{w_{1}+w_{2}}{w_{1}/\rho_{1}+w_{2}/\rho_{2}}$$

where $\rho_{1}$ and $\rho_{2}$ are the theoretical densities of LT ($\rho \;=\;3.415\;g/{cm}^{3}$) and LZT ($\rho \;=\;3.97\;g/{cm}^{3}$), respectively, and $w_{1}$ and $w_{2}$ are their corresponding mass fractions.

Rectangular bars (20 mm × 5 mm × 4 mm) were also prepared from the same granulated powders for shrinkage-curve measurements. Linear shrinkage was measured in air using a push-rod dilatometer (DIL 402PC, Netzsch-Gerätebau GmbH, Selb, Germany) at a constant heating rate of 5 °C/min. Bars of the 0.6LT–0.4LZT composition were prepared without V_2_O_5_ and with V_2_O_5_ additions (0.3 and 1.0 wt.%), using identical powder processing and forming conditions. The pressed density was controlled at ≈56% across compositions. The onset temperature of net shrinkage, $T_{onset}$, was defined as the first temperature at which the derivative $d(∆L/L_{o})/dT$ crossed zero after the initial thermal expansion. The maximum shrinkage rate temperature, $T_{peak}$, was taken as the temperature at which shrinkage rate curve $d(∆L/L_{o})/dT$ reached its most negative, corresponding to the peak shrinkage rate ($R_{peak}$).

The microstructure of the sintered specimens was examined using a field-emission scanning electron microscopy (FE-SEM, SIGMA 360, Carl Zeiss, Oberkochen, Germany). The specimens were polished with 1 μm alumina slurry and then thermally etched. The etching conditions were 1050 °C for 20 min for the base composite and 850 °C for 10 min for the V_2_O_5_-doped samples. Grain sizes were quantitatively evaluated from thermally etched SEM micrographs using ImageJ (version 1.54g) image-analysis software (NIH, Bethesda, MD, USA).

Phase identification and chemical reactivity were analyzed via powder X-ray diffraction (XRD; D/Max-2200, Rigaku, Tokyo, Japan), with Cu $K_{\alpha 1}$ radiation within a 2*θ* range of 10−70°. The diffraction patterns were collected with a step size of 0.02° (2*θ*) and a counting time of 1 s per step. To evaluate the reactivity with Ag electrodes, Ag powder was mixed with the 0.3 wt.% V_2_O_5_-doped composition powder (which exhibited the best dielectric properties) at 20 wt.% Ag, co-fired at 900 °C for 30 min, and subsequently analyzed by XRD.

The high-frequency relative permittivity ($\varepsilon_{r}$) was measured at 12 GHz using the TE_011_ mode with the Hakki–Coleman method and a network analyzer (8720ES, Agilent, Santa Clara, CA, USA). *Q × f* and $\tau_{f}$ values were measured at 9 GHz using the TE_01δ_ mode via the cavity method [29,30]. The measured $\varepsilon_{r}$ was corrected for porosity using Equation (2) [31]:

(2) $$\varepsilon_{r}^{cor}\;=\;\varepsilon_{r}^{mea}\left(1+1.5P\right)$$

where $\varepsilon_{r}^{mea}$ and $\varepsilon_{r}^{cor}$ represent the measured and corrected relative permittivity, respectively, and *P* denotes the porosity. The temperature coefficient of resonant frequency ($\tau_{f}$) was calculated using Equation (3) by measuring the resonant frequencies $f_{1}$ and $f_{2}$ at $T_{1}$ = 25 °C and $T_{2}$ = 85 °C, respectively:

(3) $$\tau_{f}\;=\;\frac{f_{2}-f_{1}}{f_{1}(T_{2}-T_{1})}$$

## 3. Results and Discussion

### 3.1. Characterization of (1 − x)Li_2.08_TiO_3_–xLi_2_ZnTi_3_O_8_ (x = 0.3–0.7) Composite Ceramics

#### 3.1.1. Sintering Behavior of (1 − x)Li_2.08_TiO_3_–xLi_2_ZnTi_3_O_8_ (x = 0.3–0.7) Composite Ceramics

**Figure 1:**
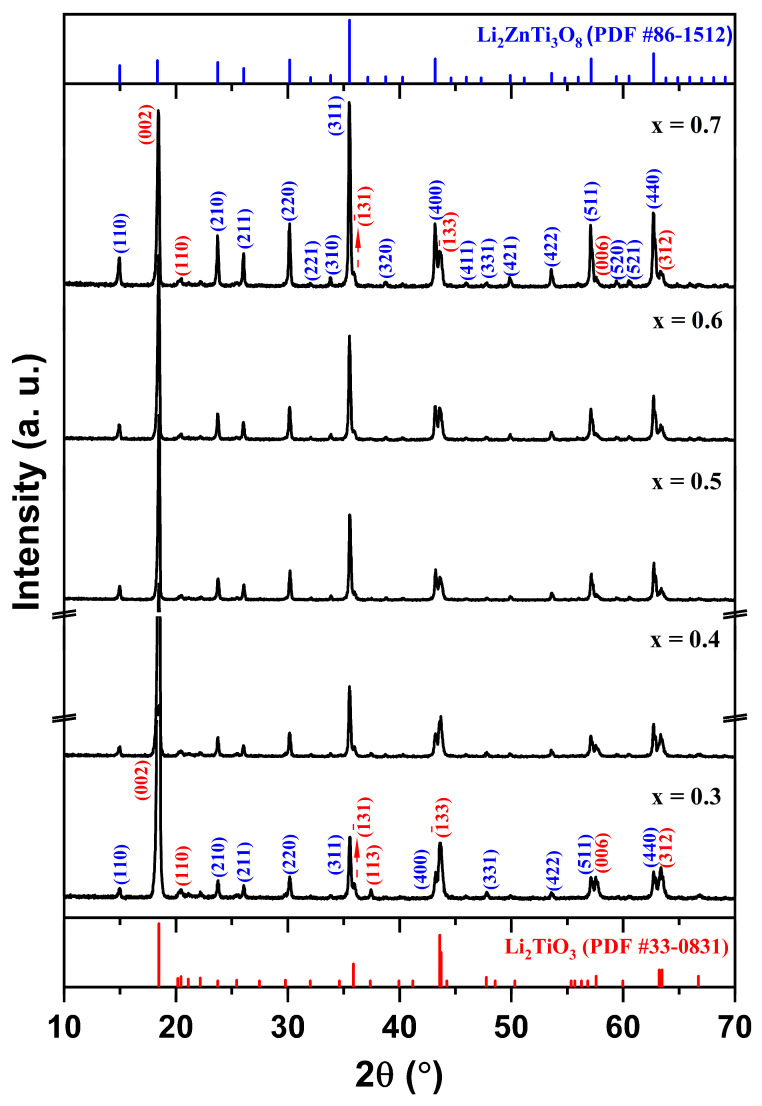
XRD patterns of (1 − x)Li_2.08_TiO_3_–xLi_2_ZnTi_3_O_8_ (x = 0.3–0.7) ceramics sintered at 1100 °C for 2 h.

Because the dielectric property averaging assumes that both phases remain structurally intact, the coexistence of two phases without any secondary reactions was verified using X-ray diffraction (XRD). Figure 1 shows the XRD patterns of (1 − x)LT–xLZT (x = 0.3–0.7) ceramics sintered at 1100 °C for 2 h. All diffraction peaks were indexed to LT (PDF #33-0831), which has a monoclinic structure (C2/c), and LZT (PDF #86-1512), which has a cubic spinel structure ($P4_{3}32$). These XRD results confirm that only the LT and LZT phases are present in the sintered specimens, with no secondary or reaction phases detected.

LT (C2/c) is reported by Bian et al. [16] to incorporate excess Li_2_CO_3_ into the lattice, forming a Li-rich solid solution. Upon heating, this Li_2_CO_3_ component can form a low-melting liquid near ~723 °C and subsequently decomposes to Li_2_O, which enhances cation mobility and facilitates incorporation into the LT framework. As a result, lithium can occupy available sites and point defects—such as cation/anion vacancies and anti-site defects—are generated to maintain charge balance. In contrast, LZT adopts a cubic spinel structure ($P4_{3}32$), with Ti and Li occupying octahedral sites and a disordered Li/Zn distribution on tetrahedral sites [32], resulting in a rigid three-dimensional framework with limited capacity to accommodate foreign cations. Because the two phases possess fundamentally different frameworks (layered monoclinic vs. cubic spinel) and because the cation-size and charge mismatch restrict mutual solubility, interdiffusion across the LT–LZT interface is expected to be extremely limited under the current sintering conditions (1100 °C, 2 h). This structural incompatibility is consistent with the absence of reaction products within the XRD detection limit and the clear coexistence of LT and LZT in the composite. Furthermore, the intensity of LZT-related peaks increases with LZT content, whereas LT maintains a strong reflection at 2θ ≈ 18° even as a minority phase (x = 0.6–0.7) due to its preferred crystallographic orientation [16,33,34].

**Figure 2:**
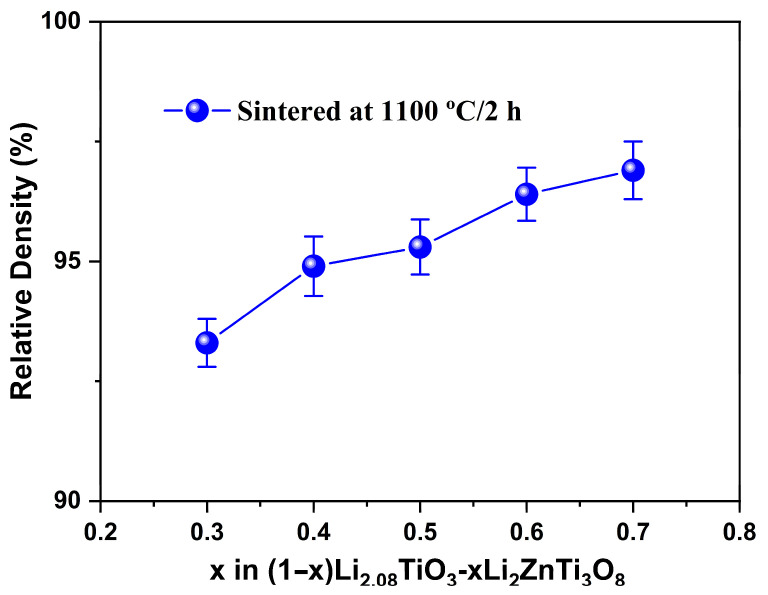
Relative sintered density of (1 − x)Li_2.08_TiO_3_–xLi_2_ZnTi_3_O_8_ (x = 0.3–0.7) ceramics sintered at 1100 °C for 2 h.

Figure 2 shows the relationship between sintered density and composition for (1 − x)LT–xLZT (x = 0.3–0.7) ceramics sintered at 1100 °C for 2 h. The sintered density increases steadily with increasing LZT fraction, demonstrating that the LZT phase plays the dominant role in governing densification in this composite system.

This trend can be rationalized in terms of the different crystal structures of the two phases. The cubic spinel LZT phase exhibits isotropic structural behavior, meaning that its grains shrink uniformly in all directions during sintering. This isotropic shrinkage more effectively eliminates surrounding pores [32]. By contrast, the LT phase, with its layered crystal structure, shows anisotropic characteristics, in which bonding strength and atomic arrangement vary with crystallographic direction. As a result, LT grains undergo anisotropic shrinkage, making densification more difficult [35]. Therefore, owing to these structural differences, increasing the LZT content progressively promotes densification in the composite.

**Figure 3:**
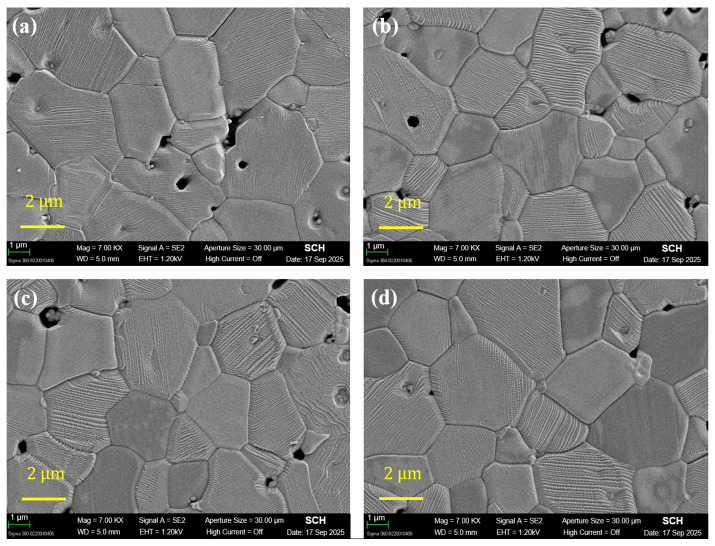
SEM images of (1 − x)Li_2.08_TiO_3_–xLi_2_ZnTi_3_O_8_ (x = 0.3–0.7) ceramics sintered at 1100 °C for 2 h: (**a**) x = 0.3; (**b**) x = 0.4; (**c**) x = 0.6; (**d**) x = 0.7.

These densification trends are consistent with the SEM microstructures in Figure 3. Figure 3a–d show the microstructures of (1 − x)LT–xLZT (x = 0.3–0.7) ceramics sintered at 1100 °C for 2 h.

As shown in Figure 3d, the LZT-rich composition exhibits markedly reduced residual porosity, consistent with more effective pore elimination expected for the isotropically shrinking LZT spinel phase. In contrast, the LT-rich composition in Figure 3a tends to retain pores at grain corners and along specific crystallographic directions, consistent with the anisotropic shrinkage behavior of the layered LT phase.

#### 3.1.2. Microwave–Dielectric Properties of (1 − x)Li_2.08_TiO_3_–xLi_2_ZnTi_3_O_8_ (x = 0.3–0.7) Composite Ceramics

**Figure 4:**
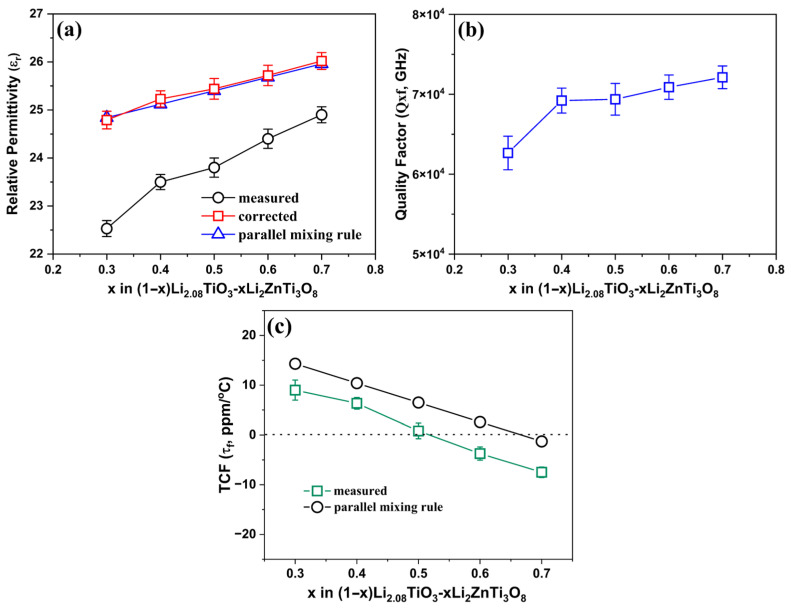
Microwave–dielectric properties of (1 − x)Li_2.08_TiO_3_–xLi_2_ZnTi_3_O_8_ (x = 0.3–0.7) ceramics sintered at 1100 °C for 2 h: (**a**) relative permittivity ($\varepsilon_{r}$); (**b**) quality factor (*Q × f*); (**c**) temperature coefficient of resonant frequency ($\tau_{f}$).

Figure 4 shows a summary of the $\varepsilon_{r}$, *Q × f*, and $\tau_{f}$ values of the (1 − x)LT–xLZT (x = 0.3–0.7) ceramics sintered at 1100 °C for 2 h. The $\varepsilon_{r}$ values were measured at 12 GHz, whereas the *Q × f* and $\tau_{f}$ values were determined at 9 GHz.

The dielectric properties of the end-member phases obtained in this study—$\;\varepsilon_{r}$ = 24.0, *Q × f* = 60,000 GHz, and $\tau_{f}$ = +26.0 ppm/°C for LT and $\varepsilon_{r}$ = 26.8, *Q × f* = 74,000 GHz, and $\tau_{f}$ = −13.0 ppm/°C for LZT—are in good agreement with previously reported values [16,17] (see Table 1).

As shown in Figure 4a, the porosity-corrected $\varepsilon_{r}^{cor}$ increases slightly as x rises from 0.3 to 0.7 and is well described by the logarithmic (Lichtenecker) mixing rule (Equation (4)):

(4) $${ln\;\varepsilon }_{r,mix}=V_{1}\ln\varepsilon_{r1}+V_{2}\ln\varepsilon_{r2}$$

where $V_{1}$ and $V_{2}$ are the volume fractions of LT and LZT, respectively, and $\varepsilon_{r1}$ and $\varepsilon_{r2}$ are their respective permittivities. This logarithmic model is commonly used for randomly intermixed two-phase microstructures [36,37]. The smooth, composition-dependent variation of $\varepsilon_{r}$ further indicates good interfacial compatibility between the LT and LZT phases and negligible Maxwell–Wagner interfacial polarization within the measured microwave frequency range [38].

As shown in Figure 4b, *Q × f* values increase from 62,600 GHz at x = 0.3 to 72,000 GHz at x = 0.7. This trend mainly reflects the increasing volume fraction of the high-Q LZT phase (*Q × f* ≈ 74,000 GHz) relative to LT (*Q × f* ≈ 60,000 GHz; Table 1), so that the composite response gradually approaches that of the LZT end member as x increases. In addition, this increase is also attributed to enhanced densification with increasing LZT content, which promotes the removal of residual porosity and thereby reduces extrinsic losses at pores and grain boundaries. This interpretation is consistent with the measured increase in relative density and with the microstructural observations (Figure 2 and Figure 3). Taken together, these results indicate that both phase composition and densification are key factors governing *Q × f* in this system, in line with previous reports on spinel–olivine composites [6] and Li_2_ATi_3_O_8_ (A = Mg, Zn) systems [17], where enhanced densification and reduced porosity are closely associated with higher *Q × f* values.

Figure 4c shows that $\tau_{f}$ values decrease nearly linearly from, +9 ppm/°C at x = 0.3 to −7.5 ppm/°C at x = 0.7, crossing zero at x ≈ 0.5. This monotonic evolution indicates effective thermal compensation between the positive-$\tau_{f}$ LT and negative-$\tau_{f}$ LZT phases. The measured $\tau_{f}$ values closely follow a simple linear-mixing behavior, consistent with earlier observations in the (1 − x)ZnAl_2_O_4_–xLi_4/3_Ti_5/3_O_4_ and (1 − x)CaWO_4_–xNa_2_WO_4_ systems [13,14].

Combining the standard identity for the temperature coefficient of resonant frequency with the definition of the temperature coefficient of permittivity leads to the linear (parallel) mixing rule shown in Equation (5):

(5) $$\tau_{f,mix}=V_{1}\tau_{f1}+V_{2}\tau_{f2}$$

This result is obtained by substituting the permittivity-mixing relation (Equation (6)) into the general identity for $\tau_{f}$ (Equation (7)):

(6) $$\tau_{Ɛ,mix}=\frac{1}{\varepsilon_{r,mix}}\frac{\left(d\varepsilon_{r,mix}\right)}{dT}=\frac{d\left(\ln\varepsilon_{r,mix}\right)}{dT}=V_{1}\tau_{\varepsilon 1}+V_{2}\tau_{\varepsilon 2}$$

(7) $$\tau_{f,mix}=-\left(\frac{1}{2}\;\tau_{\varepsilon ,mix}+\alpha_{L,mix}\right)$$

here $V_{1}$ and $V_{2}$ are the volume fractions of LT and LZT phases, respectively; $\tau_{\varepsilon }$ and $\tau_{f}$ denote the temperature coefficients of permittivity and resonant frequency; subscripts 1 and 2 refer to the LT and LZT phases; and $\alpha_{L,mix}$ is the effective linear thermal-expansion coefficient of the composite, approximated by a volume-fraction average ($\alpha_{L,mix}=\;V_{1}\alpha_{L1}+\;V_{2}\alpha_{L2}$).

A straight line computed from Equation (5) using the measured end-member $\tau_{f}$ values reproduces the overall slope, although the experimental data points are slightly offset. This modest, composition-independent offset is reasonable because residual porosity and microstructural features—such as closed pores and boundary cavities, or thin intergranular films—can still influence the temperature dependence of permittivity, $\tau_{\varepsilon }=(1/\varepsilon_{r})(d\varepsilon_{r}/dT)\;=d(ln\varepsilon_{r})/dT$, even after standard porosity correction, and consequently cause a small shift in $\tau_{f}$ [39,40]. In addition, Equation (7) indicates that the thermal-expansion term can act as a constant offset: minor departures of the linear expansion coefficient ($\alpha_{L}$)—arising from CTE mismatch and mechanical constraint between the layered LT and spinel LZT phases, or small residual thermal-expansion stresses at their interfaces—will further shift $\tau_{f}$. These factors explain why the LT–LZT data exhibit an essentially linear dependence on x, consistent with two-phase mixing, yet display a small systematic deviation from the ideal parallel mixing prediction.

Overall, the (1 − x)LT–xLZT series delivers a well-balanced combination of moderate permittivity ($\varepsilon_{r}$ = 24.79–26.02), high quality factor (*Q × f* > 62,000 GHz), and near-zero $\tau_{f}$ at x ≈ 0.5, satisfying the key requirements for low-loss microwave–dielectrics in LTCC applications. These results indicate that the mutual phase stability between the layered LT and spinel LZT enables precise tuning of dielectric properties through composition control, consistent with design principles reported for recent multiphase microwave–dielectric composites [13,14,15]. Collectively, the ability to systematically tune $\tau_{f}$ while maintaining a high *Q × f* demonstrates that composite engineering is an effective strategy for realizing temperature-stable, low-dielectric-loss microwave ceramics.

Nevertheless, the present series still requires a sintering temperature of 1100 °C—exceeding the ≤950 °C LTCC processing limit—so that next section explores a V_2_O_5_-assisted approach to reduce the firing temperature of (1 − x)LT–xLZT while preserving its microwave performance.

### 3.2. V_2_O_5_-Assisted Low-Temperature Sintering of 0.6Li_2.08_TiO_3_–0.4Li_2_ZnTi_3_O_8_: Densification, Microstructure, and Microwave–Dielectric Properties

Within the undoped series, x = 0.5 yields a near-zero $\tau_{f}$; however, for LTCC applications, we adopt 0.6LT–0.4LZT as the base composition because its slightly positive $\tau_{f}$ provides a margin to accommodate the negative $\tau_{f}$ shift typically induced by V_2_O_5_, with minimal degradation of $\varepsilon_{r}$ and *Q × f*. Accordingly, in this section we explore a V_2_O_5_-assisted route to sub-950 °C co-firing by examining (i) sintering behavior and microstructure when sintered at 900 °C for 30 min, (ii) SEM/EDS evidence for liquid-phase sintering, (iii) the resulting microwave–dielectric properties, and (iv) Ag co-firing compatibility.

#### 3.2.1. Sintering Behavior and Microstructure of V_2_O_5_-Doped 0.6LT–0.4LZT Ceramics

To satisfy the ≤950 °C co-firing requirement for LTCC, we aimed to reduce the 1100 °C sintering temperature of the 0.6LT–0.4LZT composite. To this end, V_2_O_5_ (melting point ≈ 690 °C), a well-known sintering aid for LTCC dielectrics, was introduced [21,22,23,24,25,26,27,28].

**Figure 5:**
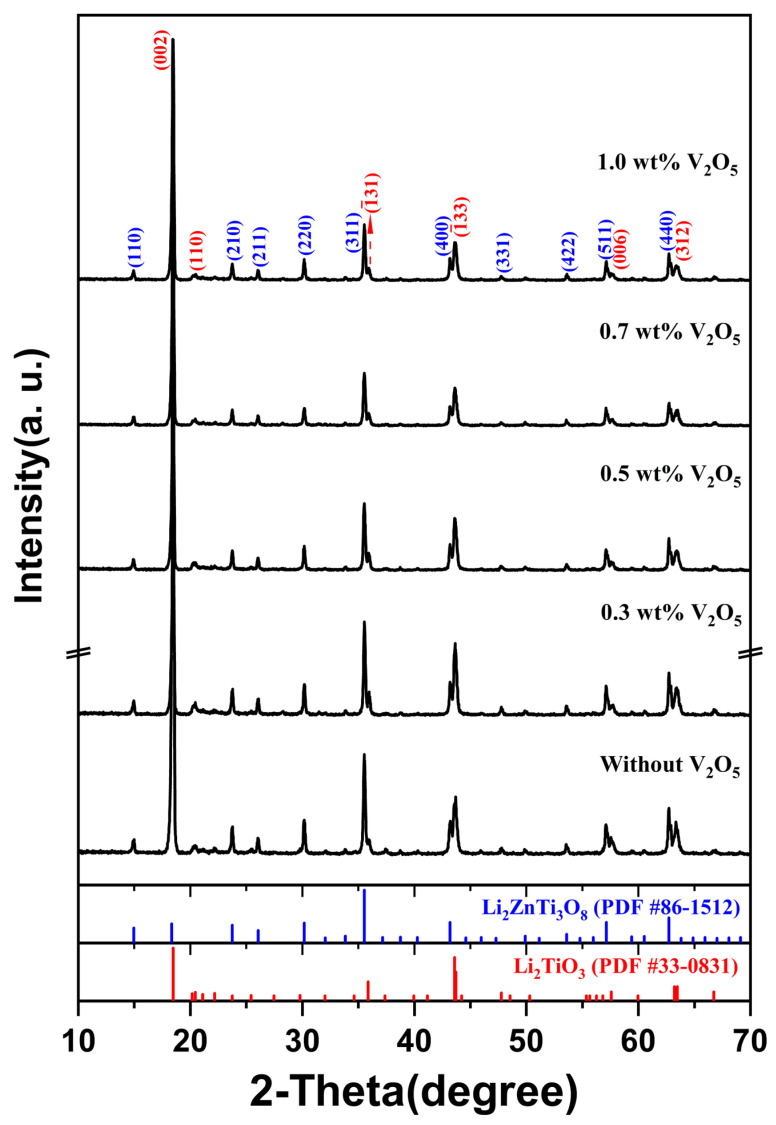
XRD patterns of 0.6Li_2.08_TiO_3_–0.4Li_2_ZnTi_3_O_8_ ceramics containing 0.3–1.0 wt.% V_2_O_5_ and sintered at 900 °C for 30 min. The XRD pattern of the undoped 0.6Li_2.08_TiO_3_–0.4Li_2_ZnTi_3_O_8_ ceramic sintered at 1100 °C for 2 h is also shown as a reference to confirm the absence of reaction phases.

As a prerequisite for reliable evaluation of material properties, we first verified phase stability under LTCC-relevant firing conditions. Figure 5 shows XRD patterns of 0.6LT–0.4LZT doped with 0.3–1 wt.% V_2_O_5_ and sintered at 900 °C for 30 min. Within the XRD detection limit, only reflections from LT and LZT are observed; no reaction products are detected. This confirmation ensures that the subsequent microwave–dielectric properties are not confounded by secondary phases formed during sintering.

**Figure 6:**
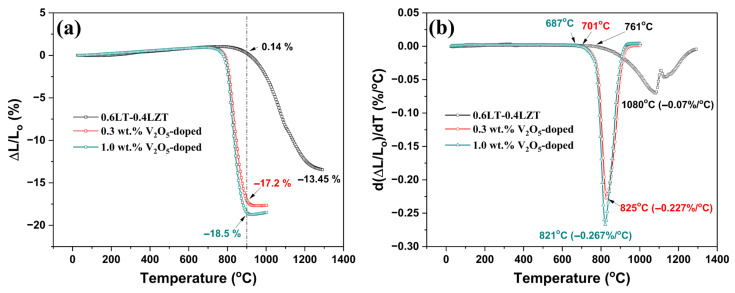
Dilatometry of 0.6Li_2.08_TiO_3_–0.4Li_2_ZnTi_3_O_8_ with and without V_2_O_5_. (**a**) Linear shrinkage, $∆L/L_{o}$; (**b**) shrinkage rate, $d(\Delta L/L_{o})/dT$.

Having established that V_2_O_5_ additions do not generate XRD-detectable reaction products under the chosen sintering schedule, we next evaluated densification kinetics by dilatometry to determine the onset and peak temperatures of shrinkage. These kinetic markers define the practical sintering window and frame the interpretation of the microstructural signatures observed at 900 °C/30 min. Figure 6 presents the dilatometry responses of 0.6LT–0.4LZT with and without V_2_O_5_ additions.

At 900 °C, the undoped sample exhibits essentially no net shrinkage, whereas the 0.3 and 1.0 wt.% V_2_O_5_-doped specimens show large shrinkages $\left(∆L/L_{o}\right)$ of −17.2% and −18.5%, respectively. The doped curves also show crossover from thermal expansion to net shrinkage much earlier ($T_{onset}$ ≈ 680–700 °C), while the undoped specimen remains in the expansion regime to a substantially higher temperature ($T_{onset}$ ≈ 760 °C). This pronounced difference in $∆L/L_{o}$ at 900 °C and the earlier $T_{onset}$ provide strong quantitative evidence for V_2_O_5_-assisted liquid-phase sintering, together with the large reductions in $T_{peak}$ and the increase in $R_{peak}$: the V_2_O_5_-free sample exhibits a rate maximum at $T_{peak}$ ≈ 1080 °C with $R_{peak}$ ≈ −0.07%/°C, whereas 0.3 and 1.0 wt.% V_2_O_5_ shift $T_{peak}$ to ≈825 °C and ≈821 °C and increase the $R_{peak}$ to ≈−0.227%/°C and ≈−0.267%/°C, respectively. Notably, the 0.3 and 1.0 wt.% curves nearly overlap across both $∆L/L_{o}$ and $d(\Delta L/L_{o})/dT$, indicating that ≈0.3 wt.% V_2_O_5_ is sufficient to enter the low-temperature densification regime and that higher contents provide little additional kinetic benefit.

**Figure 7:**
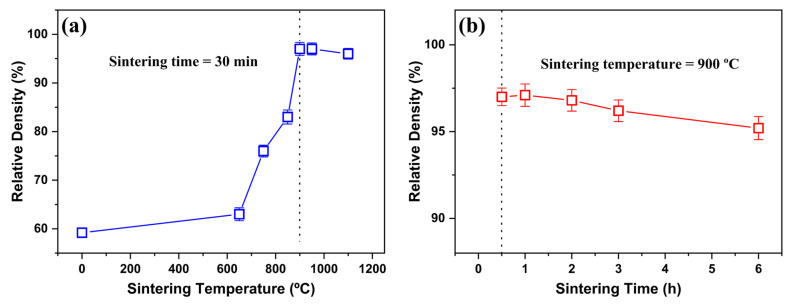
Effect of 0.3 wt.% V_2_O_5_ addition on the sintering behavior of 0.6Li_2.08_TiO_3_–0.4Li_2_ZnTi_3_O_8_ ceramics: (**a**) relative density versus sintering temperature at a fixed dwell of 30 min; (**b**) relative density versus dwell time.

**Figure 8:**
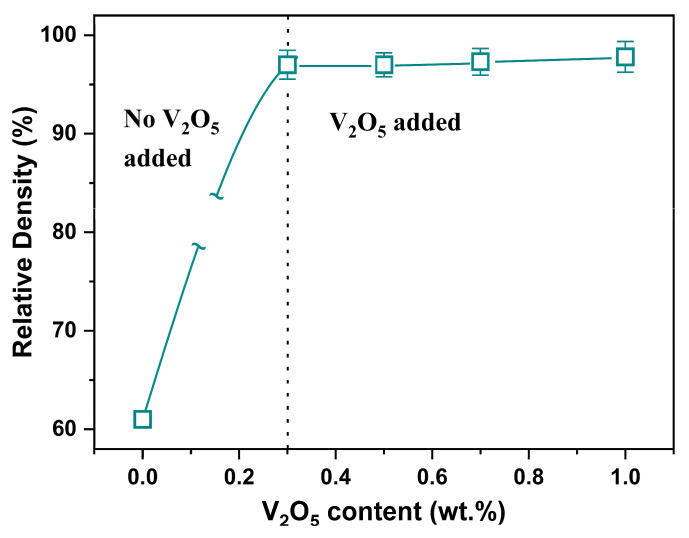
Effect of V_2_O_5_ addition on the relative density of 0.6Li_2.08_TiO_3_–0.4Li_2_ZnTi_3_O_8_ ceramics sintered at 900 °C for 30 min. The vertical dashed line at 0.3 wt.% marks the onset of V_2_O_5_ addition.

**Figure 9:**
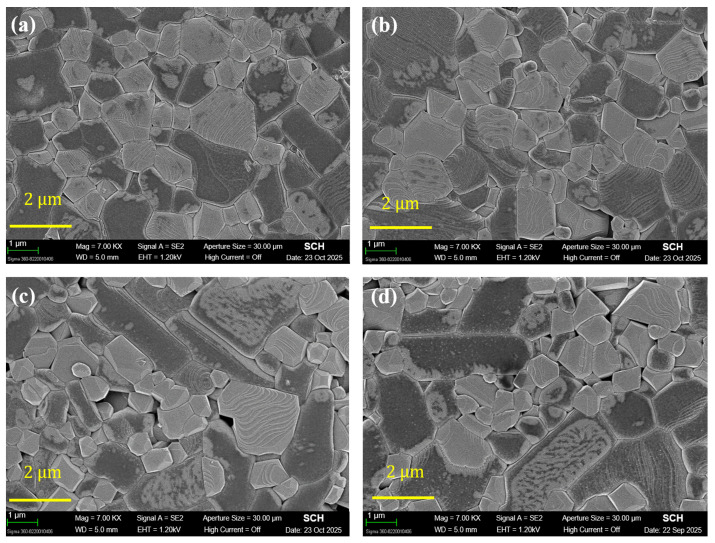
SEM images of V_2_O_5_-doped 0.6Li_2.08_TiO_3_–0.4Li_2_ZnTi_3_O_8_ ceramics sintered at 900 °C for 30 min: (**a**) 0.3 wt.%; (**b**) 0.5 wt.%; (**c**) 0.7 wt.%; (**d**) 1.0 wt.% V_2_O_5_ addition.

**Figure 10:**
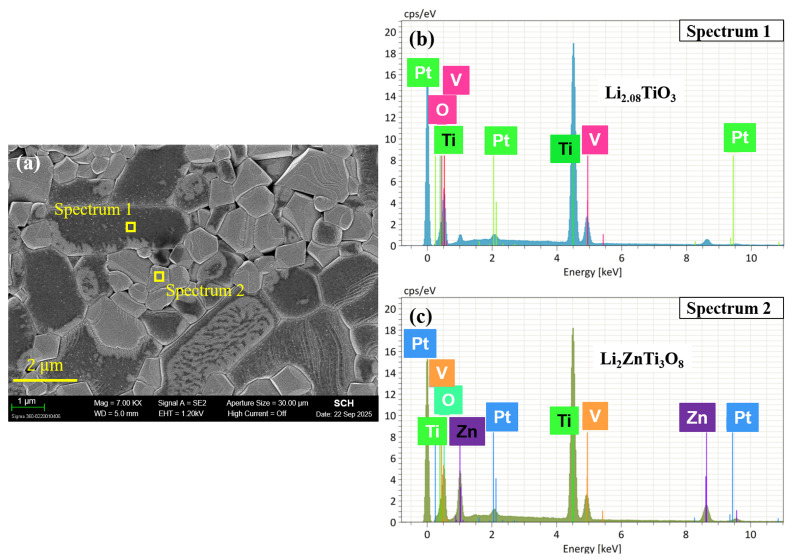
SEM micrograph and corresponding EDS analyses of 1.0 wt.% V_2_O_5_-doped 0.6Li_2.08_TiO_3_–0.4Li_2_ZnTi_3_O_8_ ceramics sintered at 900 °C for 30 min: (**a**) SEM image showing the spot analyzed via EDS; (**b**) EDS spectrum of a coarsened Li_2.08_TiO_3_ grain; (**c**) EDS spectrum of a non-coarsened Li_2_ZnTi_3_O_8_ grain.

**Figure 11:**
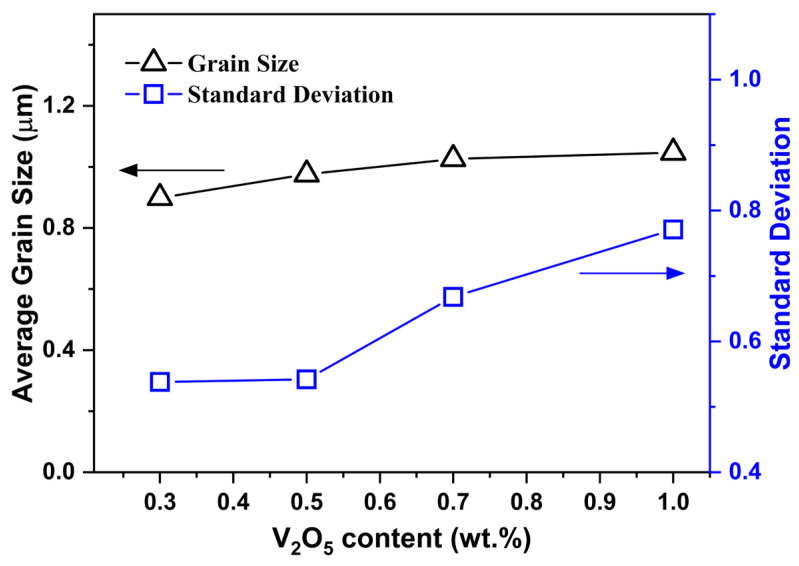
Grain size and standard deviation as a function of V_2_O_5_ content (0.3–1.0 wt.%) for 0.6Li_2.08_TiO_3_–0.4Li_2_ZnTi_3_O_8_ ceramics sintered at 900 °C for 30 min. The corresponding grain-size distribution histograms are provided in Supplementary Figure S1.

These kinetic signatures align with three independent observations presented later (Figure 7, Figure 8, Figure 9, Figure 10 and Figure 11): (i) near-full density at 900 °C/30 min (≈97% relative density; Figure 7), (ii) a densification plateau with increasing V_2_O_5_ content (Figure 8), and (iii) rounded grain edges and LT-selective abnormal grain growth at 900 °C/30 min (Figure 9, Figure 10 and Figure 11). Taken together, these dilatometry signatures—an early onset of net shrinkage, an early rate peak, large total shrinkage at 900 °C, and a higher peak shrinkage rate ($R_{peak}$)—are consistent with a V_2_O_5_-related liquid-phase sintering behavior in this multicomponent system.

Exact coincidence of $T_{onset}$ with the melting point of pure V_2_O_5_ (~690 °C) is not expected; in multicomponent systems the effective liquidus, wetting, and flow behavior can deviate from those of the pure oxide and depend on the applied heating rate. The near overlap of the 0.3 and 1.0 wt.% curves further rationalizes the explanation for why densification saturates at the lowest addition, while excess V_2_O_5_ primarily broadens the LT grain-size distribution and increases interfacial losses, consistent with the gradual decrease in *Q × f* at contents above 0.3 wt.%.

Accordingly, we optimized the sintering condition for the 0.3 wt.% V_2_O_5_-doped 0.6LT–0.4LZT composition using $T_{onset}$ and $T_{peak}$ as practical bounds. Figure 7a shows the temperature dependence of the sintered density for 0.6LT–0.4LZT containing 0.3 wt.% V_2_O_5_ at a fixed dwell of 30 min. The green compact exhibited an initial relative density of 59.2%, which increased progressively with temperature, reaching 97% at 900 °C, confirming successful densification. A further increase in temperature above 900 °C slightly decreased the density to 96%.

Figure 7b shows the time dependence of densification at 900 °C. The relative density reached a maximum at 30 min, and extended dwell times did not provide additional benefits. Collectively, these results establish that 900 °C for 30 min is the optimal sintering condition for the 0.3 wt.% V_2_O_5_-doped composition. Notably, a trace V_2_O_5_ addition reduces the temperature for near-full densification by ≈200 °C (from 1100 to 900 °C) and shortens dwell time from 2 h to 30 min, consistent with the kinetic shift revealed by dilatometry.

With the sintering schedule fixed at 900 °C for 30 min, we next assessed how V_2_O_5_ content affects densification under this window. Figure 8 summarizes the relative density of 0.6LT–0.4LZT specimens sintered at 900 °C for 30 min as a function of V_2_O_5_ content (0–1.0 wt.%).

The break in the connecting line between 0 and 0.3 wt.% indicates that no compositions were measured in this interval. Under these conditions, the relative density increases from 61% (0 wt.% V_2_O_5_) to 97% at 0.3 wt.%, and remains essentially constant (within experimental scatter) at 97.8% up to 1.0 wt.%, indicating a densification plateau. As shown by the SEM/EDS and grain-size statistics (Figure 9, Figure 10 and Figure 11), V_2_O_5_ contents above 0.3 wt.% promote LT-selective, non-uniform grain coarsening, which yields little benefit for densification and increases the risk of microstructural heterogeneity. Accordingly, 0.3 wt.% V_2_O_5_ is identified as the practical optimum, achieving near-full density at 900 °C for 30 min without compromising microstructural uniformity or subsequent microwave–dielectric performance. This densification behavior aligns with the expected liquid-phase sintering kinetics reported for V_2_O_5_-doped systems [21,22,23,24,25,26,27,28] and delineates a practical LTCC co-firing window at 900 °C for 30 min.

Figure 9 shows the SEM microstructures of 0.6LT–0.4LZT ceramics doped with 0.3–1.0 wt.% V_2_O_5_ and sintered at 900 °C for 30 min. Progressive rounding of grain edges is evident, and the fraction of abnormally coarsened grains increases with V_2_O_5_ content. These features are characteristics of liquid-phase sintering, where a V_2_O_5_-derived liquid can wet boundaries and accelerate solution-reprecipitation, consistent with prior reports on V_2_O_5_-based fluxes [21,22,23,24,25,41,42].

The microstructural signatures are corroborated by dilatometry, which provides process-level evidence for liquid-assisted sintering, with $T_{peak}$ reduced by about 250 °C relative to the undoped ceramic (Figure 6). No crystalline reaction products are detected by XRD (Figure 5), which is reasonable at this trace additive level and given the limited sensitivity of XRD to minor or poorly crystalline vanadate boundary species. Although direct identification of any boundary liquid is beyond the present scope, the combined dilatometry and SEM evidence supports a liquid-phase-assisted densification mechanism.

The microstructural signatures in Figure 9, particularly the progressive rounding of grain edges and the emergence of abnormally coarsened grains at higher V_2_O_5_ levels, suggest the involvement of a liquid phase during sintering. To identify which phase undergoes abnormal coarsening, compositional analysis was conducted. Spot-EDS performed on representative grains (Figure 10), together with the grain-type classification from morphology, indicate that the abnormally coarsened grains correspond to LT, while the finer or non-coarsened grains correspond to LZT. This establishes that the abnormal grain growth induced by V_2_O_5_ is phase-selective and occurs predominantly in LT grains.

Consistent with this phase selectivity, Figure 11 presents the quantitative evolution of grain-size statistics for V_2_O_5_-doped 0.6LT–0.4LZT ceramics sintered at 900 °C for 30 min. Although the average grain size increases only modestly with increasing V_2_O_5_ content, the standard deviation rises sharply for V_2_O_5_ ≥ 0.7 wt.%, indicating a substantially broadened grain-size distribution driven by LT-selective abnormal coarsening. This statistical widening aligns with the SEM observations in Figure 9 and confirms a progressive loss of microstructural uniformity at elevated V_2_O_5_ levels.

Together, these findings—rounded grains, LT-selective abnormal coarsening, and broadening of the grain-size distribution—form a coherent set of microstructural signatures characteristic of liquid-phase-assisted sintering.

Accordingly, the grain-growth response is phase selective, with LT experiencing pronounced coarsening while LZT shows only limited growth. A plausible interpretation is that a low-viscosity, V_2_O_5_-derived liquid forms at intermediate temperatures during firing. In this liquid environment, the two phases interact asymmetrically—LT experiencing more pronounced dissolution–reprecipitation pathways, whereas the more rigid spinel LZT is far less affected—consistent with the observed LT-selective abnormal grain growth. The layered LT phase is more susceptible to liquid-mediated mass transport, whereas the three-dimensionally bonded spinel LZT exhibits limited solubility and consequently limited grain growth. A similar mechanism has been documented for V_2_O_5_-doped layered Li–Nb–Ti oxides [28], where the layered framework dissolves into V_2_O_5_-based liquids and reprecipitates as abnormally coarsened grains. In this context, V_2_O_5_-assisted liquid-phase sintering not only promotes densification (≥97% relative density) but also accelerates mass transport that drives LT-selective abnormal grain growth, as evidenced by Figure 9, Figure 10 and Figure 11.

#### 3.2.2. Microwave–Dielectric Properties of V_2_O_5_-Doped 0.6LT–0.4LZT Ceramics

**Figure 12:**
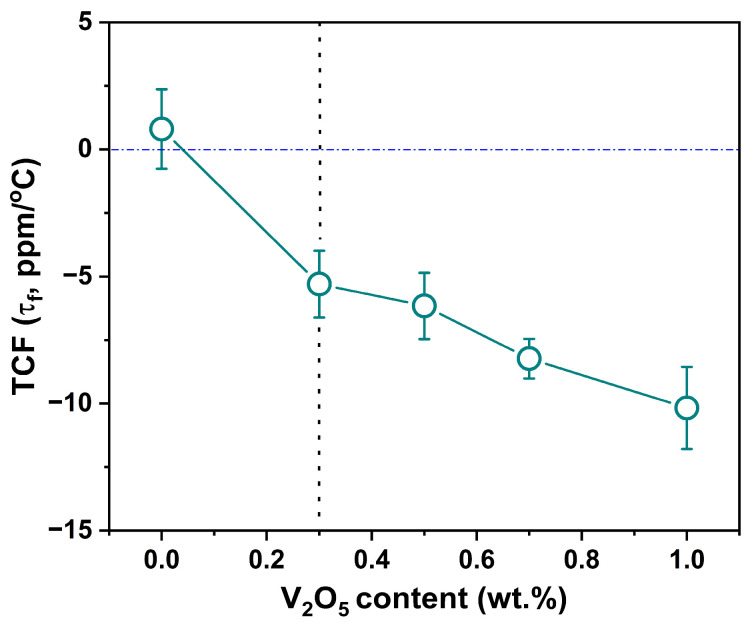
Effect of V_2_O_5_ addition on the temperature coefficient of resonant frequency ($\tau_{f}$) for 0.5Li_2.08_TiO_3_–0.5Li_2_ZnTi_3_O_8_ ceramics. The $\tau_{f}$ at 0 wt.% data point was measured when sintered at 1100 °C for 2 h, while the $\tau_{f}$ at the V_2_O_5_-doped data points at 900 °C for 30 min. The vertical dashed line at 0.3 wt.% marks the onset of V_2_O_5_ addition.

**Figure 13:**
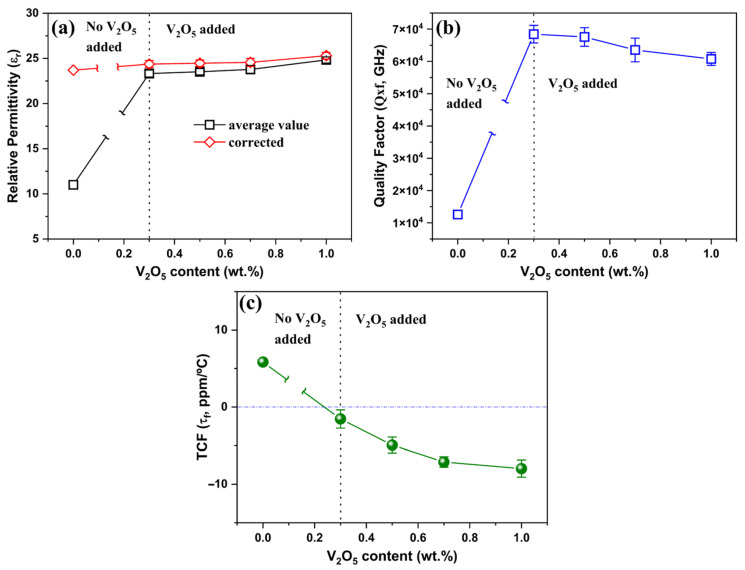
Effect of V_2_O_5_ addition on the microwave–dielectric properties of 0.6Li_2.08_TiO_3_–0.4Li_2_ZnTi_3_O_8_ ceramics sintered at 900 °C for 30 min: (**a**) relative permittivity ($\varepsilon_{r}$); (**b**) quality factor (*Q × f*); (**c**) temperature coefficient of resonant frequency ($\tau_{f}$). The vertical dashed line at 0.3 wt.% indicates the onset of V_2_O_5_ addition.

As a reference, Figure 12 first shows $\tau_{f}$ tuning in the equimolar composite (0.5LT–0.5LZT), and Figure 13 then shows the full dielectric response of the base composition (0.6LT–0.4LZT) with V_2_O_5_.

Figure 12 shows the change in $\tau_{f}$ for the 0.5LT−0.5LZT composite with 0.3–1.0 wt.% V_2_O_5_, measured at 9 GHz. For the equimolar composition sintered at 1100 °C for 2 h, $\tau_{f}$ is +0.8 ppm/°C, representing the most temperature-stable member of the undoped LT–LZT series. With increasing V_2_O_5_ content (900 °C/30 min), $\tau_{f}$ shifts progressively toward more negative values, reaching −5.53 ppm/°C at 0.3 wt.%, and becomes more negative with further additions. Thus, even a minimal V_2_O_5_ addition is sufficient to perturb $\tau_{f}$ to the negative side of zero, in qualitative agreement with prior observations in other V_2_O_5_-containing microwave–dielectrics [25,26,27].

In this context, the equimolar data shown in Figure 12 provide a reference point for the V_2_O_5_-induced negative shift in $\tau_{f}$, while the base composition (0.6LT–0.4LZT) used in this study offers a modest positive $\tau_{f}$ baseline that can be tuned toward zero (Figure 13). Figure 13 presents the dielectric properties ($\varepsilon_{r}$, *Q × f*, and $\tau_{f}$) of V_2_O_5_-doped 0.6LT–0.4LZT as a function of V_2_O_5_ content, measured at 12 and 9 GHz.

Figure 13a shows the measured ($\varepsilon_{r}^{mea}$) and porosity-corrected ($\varepsilon_{r}^{cor}$) relative permittivity values of 0.6LT–0.4LZT as a function of V_2_O_5_ content (0.3–1.0 wt.%). All samples including the undoped (0 wt.% V_2_O_5_) composition were sintered under identical conditions at 900 °C for 30 min. The measured $\varepsilon_{r}^{mea}$ increases sharply from 11.2 at 0 wt.% to 23.32–24.84 at ≥0.3 wt.%, reflecting substantial V_2_O_5_-induced densification at 900 °C, as corroborated by the density data in Figure 8. Accordingly, the rise in $\varepsilon_{r}$ is primarily attributed to reduced residual porosity, consistent with the well-known porosity sensitivity of ceramic dielectrics [39,40]. Within this V_2_O_5_ range, the permittivity remains nearly constant, exhibiting only minor fluctuations without a clear systematic trend once a high relative density (~97%) is achieved. For the V_2_O_5_-free 0.4LT–0.6LZT specimen sintered at 900 °C for 30 min ($\varepsilon_{r}^{mea}$ = 11.0, P = 0.39), back-calculation using the symmetric Bruggeman effective-medium relation yields a dense-body permittivity of $\varepsilon_{r}^{cor}$ = 23.7. This value agrees well with the porosity-corrected $\varepsilon_{r}^{cor}$ = 25.23 obtained for the same composition after sintering at 1100 °C for 2 h, indicating that the intrinsic permittivity of fully dense (1 − x)LT–xLZT lies within a narrow range of $\varepsilon_{r}$ = 24–26, essentially independent of V_2_O_5_ addition. Consequently, the $\varepsilon_{r}^{cor}$ values for all compositions, including the 0 wt.% V_2_O_5_-doped sample, cluster near the intrinsic level, confirming that V_2_O_5_ primarily enhances densification and affords $\tau_{f}$ tunability, with minimal effect on the inherent dielectric polarizability of the composite.

Figure 13b shows *Q × f* as a function of V_2_O_5_ content, measured at 9 GHz. The undoped base composition exhibits a low value of 12,600 GHz, whereas 0.3 wt.% V_2_O_5_ increases *Q × f* to 68,400 GHz, comparable to the value (69,200 GHz) obtained for the base composition under the original high-temperature sintering. This sharp increase at 0.3 wt.% V_2_O_5_ is primarily attributable to densification achieved at 900 °C. Practically, these results delineate a processing window: 0.3 wt.% V_2_O_5_ maximizes *Q × f* at 900 °C for 30 min, whereas further additions (V_2_O_5_ > 0.3 wt.%) introduce microstructure-related interfacial losses that outweigh the modest gains from reduced grain-boundary area; accordingly, *Q × f* gradually decreases with further V_2_O_5_. Herein, two competing effects govern the evolution of *Q × f* in this system: (i) an enhancement in *Q × f* resulting from grain coarsening, which reduces the total grain-boundary area [43,44], and (ii) a degradation in *Q × f* that is commonly observed when liquid-phase sintering additives are present in excessive amounts. A similar decrease in *Q × f* with increasing V_2_O_5_ content has been reported in other V_2_O_5_-assisted liquid-phase sintering systems [26,28]. In the present composite, the observed decline in *Q × f* beyond 0.3 wt.% can be reasonably associated with the microstructural non-uniformity induced at higher V_2_O_5_ levels—most notably LT-selective abnormal grain coarsening and grain-size-distribution broadening (Figure 9 and Figure 11). This microstructural heterogeneity increases dielectric loss through interfacial scattering and local field mismatch, thereby lowering *Q × f* [45,46]. In addition, potential dielectric losses associated with V-based species at higher V_2_O_5_ levels cannot be ruled out and may also contribute to the observed reduction in *Q × f*.

Figure 13c illustrates the variation in $\tau_{f}$ for 0.6LT–0.4LZT as a function of V_2_O_5_ content (0.3–1.0 wt.%). The undoped 0.6LT–0.4LZT sample, despite its relatively low density of 61% (Figure 8), exhibits a $\tau_{f}$ value of +5.84 ppm/°C, only slightly lower than that of the fully dense specimen (+6.35 ppm/°C, Figure 4). This weak sensitivity of $\tau_{f}$ to porosity contrasts with the strong porosity dependence observed for $\varepsilon_{r}$ and *Q × f* (Figure 13a,b) and is consistent with previous reports indicating that extrinsic microstructural features, such as pores, have a much greater influence on permittivity and dielectric-loss than on $\tau_{f}$ itself [39]. Upon V_2_O_5_ addition, $\tau_{f}$ for 0.6LT–0.4LZT shifts steadily toward more negative values, mirroring the behavior observed for the 0.5LT–0.5LZT composition in Figure 12. Increasing V_2_O_5_ content from 0 to 1.0 wt.% drives $\tau_{f}$ from the positive region (+5.84 ppm/°C) into the negative region, with $\tau_{f}$ = −4.93 ppm/°C at 0.5 wt.% and −7.97 ppm/°C at 1.0 wt.%. The composition containing 0.3 wt.% V_2_O_5_ is particularly noteworthy: $\tau_{f}$ is reduced to −1.55 ppm/°C, a value much closer to zero than that of the 0.5LT–0.5LZT reference. This result indicates that this small amount of V_2_O_5_ is sufficient to shift the $\tau_{f}$ toward near-zero compensation without compromising $\varepsilon_{r}$ or *Q × f*.

#### 3.2.3. Ag Co-Firing Compatibility of 0.3 wt.% V_2_O_5_-Doped 0.6LT–0.4LZT Ceramic

**Figure 14:**
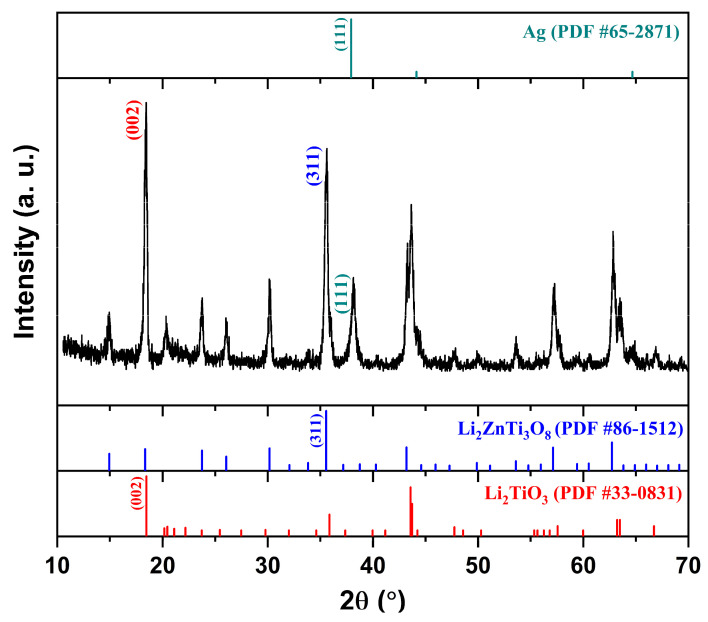
XRD patterns of 0.3 wt.% V_2_O_5_-doped 0.6Li_2.08_TiO_3_–0.4Li_2_ZnTi_3_O_8_ ceramics mixed with 20 wt.% Ag and co-fired in air at 900 °C for 30 min.

To assess Ag electrode compatibility, the 0.3 wt.% V_2_O_5_-doped 0.6LT–0.4LZT composition—which exhibited the highest *Q × f* and a near-zero $\tau_{f}$ at 900 °C for 30 min—was mixed with 20 wt.% Ag powder and co-fired at 900 °C for 30 min in air, matching the dielectric sintering schedule. The XRD pattern in Figure 14 shows only reflections of Li_2.08_TiO_3_, Li_2_ZnTi_3_O_8_, and metallic Ag, and no additional reaction products are detected within the XRD detection limit. This indicates that under LTCC-relevant conditions, the dielectric is chemically compatible with Ag.

From a practical LTCC standpoint, the performance of the present composite may be compared with two relevant groups of composite dielectrics reported in the literature. The first group comprises ceramic–ceramic composite LTCC dielectrics with moderate permittivity ($\varepsilon_{r}$ ≈ 20–30), such as CaTiO_3_–CaZrO_3_-based systems, which can achieve near-zero $\tau_{f}$ but often rely on glass frits and tend to exhibit lower *Q × f* values [47]. The second group includes systems where V_2_O_5_ serves as a low-temperature sintering aid (e.g., V_2_O_5_-added SrTiO_3_–La(Mg_0.5_Ti_0.5_)O_3_ or MgTiO_3_), in which densification is enhanced but the resulting dielectric performance is generally insufficient for high-Q LTCC applications, and Ag co-firing compatibility is seldom reported [21,22].

A related LT–LZT composition processed with 0.75 wt.% LBSCA glass (0.73LZT–0.27LT) achieved $\varepsilon_{r}$ ≈ 23.9, *Q × f* ≈ 63,050 GHz, and $\tau_{f}$ ≈ +1.2 ppm/°C at 900 °C for 3 h. While these results confirm that near-zero $\tau_{f}$ is attainable in LT–LZT, the longer sintering time and lack of Ag compatibility data limit its LTCC applicability [15].

In contrast, the optimized 0.6LT–0.4LZT composition with 0.3 wt.% V_2_O_5_ developed in this study achieves a balanced combination of moderate permittivity ($\varepsilon_{r}$ ≈ 23.3), high *Q × f* (≈68,400 GHz), and near-zero $\tau_{f}$ (−1.55 ppm/°C), together with densification at 900 °C within 30 min and confirmed Ag co-firing compatibility, placing it among competitive LTCC dielectric candidates.

## 4. Conclusions

The newly developed (1 − x)LT–xLZT composite series exhibits excellent intrinsic microwave–dielectric performance but requires a high sintering temperature (1100 °C) in the undoped state, limiting LTCC compatibility. To overcome this limitation, 0.6Li_2.08_TiO_3_−0.4Li_2_ZnTi_3_O_8_ containing 0.3–1.0 wt.% V_2_O_5_ was successfully densified at 900 °C for 30 min, achieving a relative density above 97% while maintaining the coexistence of the LT and LZT phases without any XRD-detectable reaction products. Microstructural analysis indicates that V_2_O_5_ acts as a liquid former in the 0.6LT–0.4LZT composition, promoting liquid-phase sintering and LT-selective grain coarsening. Complementary dilatometry provides process-level evidence for this V_2_O_5_-assisted liquid-phase sintering mechanism: doped samples show large total shrinkage at 900 °C ($∆L/L_{o}$ = −17.2 to −18.5%), an early onset ($T_{onset}$ ≈ 680–700 °C) and early rate peak ($T_{peak}$ ≈ 821–825 °C vs. 1080 °C undoped), together with a higher peak shrinkage rate ($R_{peak}$ magnitude ≈ 0.23–0.27%/°C vs. 0.07%/°C). The near overlap of the 0.3 and 1.0 wt.% curves indicates that 0.3 wt.% is sufficient, supporting 900 °C/30 min as the practical firing window.

At 0.3 wt.% V_2_O_5_, the composition exhibits $\varepsilon_{r}$ = 23.32, *Q × f* = 68,400 GHz, and $\tau_{f}$ = −1.55 ppm/°C at 900 °C/30 min—representing near-intrinsic permittivity, a high-quality factor comparable to the high-temperature baseline, and a $\tau_{f}$ value close to zero. Further increases in V_2_O_5_ (>0.3 wt.%) cause a gradual decrease in *Q × f*, due to excess-liquid–induced microstructural non-uniformity. In particular, V_2_O_5_ promotes phase-selective abnormal coarsening of LT grains, which broadens the overall grain-size distribution and increases interfacial losses.

Ag co-firing tests confirm electrode compatibility under LTCC-relevant conditions (900 °C for 30 min), with no additional phases detected by XRD after mixing with 20 wt.% Ag.

In summary, sintering with 0.3 wt.% V_2_O_5_ at 900 °C for 30 min provides a practical processing window that meets all key LTCC requirements—sub-950 °C firing, moderate $\varepsilon_{r}$, high *Q × f*, near-zero $\tau_{f}$, and Ag compatibility—establishing the V_2_O_5_-assisted 0.6LT–0.4LZT dielectric as a strong candidate for temperature-stable, low-loss LTCC components for 5G and next-generation (5G-advanced/6G) microwave applications.

## Figures and Tables

**Table 1:** Comparison of the dielectric properties ($\varepsilon_{r}$, *Q × f*, and $\tau_{f}$) of Li_2.08_TiO_3_ and Li_2_ZnTi_3_O_8_ ceramics in this study with the literature.

Composition	Reference	$\varepsilon_{r}$	Q × f (GHz)	$\tau_{f}$ (ppm/°C)
Li_2.08_TiO_3_	This study	24.0	60,000	+26.0
	Bian et al. [16]	24.6	66,000	+22.1
Li_2_ZnTi_3_O_8_	This study	26.8	74,000	−13.0
	George et al. [17]	25.6	72,000	−11.22

## Data Availability

The original contributions presented in this study are included in the article/Supplementary Materials. Further inquiries can be directed to the corresponding author.

## Supplementary Materials

The following supporting information can be downloaded at: https://www.mdpi.com/article/10.3390/ma19010094/s1, Supplementary Figure S1. Grain-size distribution histograms (probability density) for the 0.6LT–0.4LZT composite sintered with different V_2_O_5_ contents: (a) 0.3 wt.%, (b) 0.5 wt.%, (c) 0.7 wt.%, and (d) 1.0 wt.%. All histograms are plotted using a common grain-size axis to enable direct comparison. With increasing V_2_O_5_ content, the distributions progressively broaden and develop a pronounced high-size tail, while the central part of the distribution does not shift significantly. This behavior indicates the emergence of a minor population of abnormally coarsened LT grains rather than a uniform shift of the entire grain-size distribution.
