# Supplementary material for: V2O5-Assisted Low-Temperature Sintering and Microwave Dielectric Properties of (1 − x)Li2.08TiO3–xLi2ZnTi3O8 (x = 0.3−0.7) Ceramics for LTCC Applications
Source: Materials (Basel). 2025 Dec 26;19(1):94. doi: 10.3390/ma19010094 (PMC12787004; doi:10.3390/ma19010094)
Supplement: Supplementary file 1 [file materials-19-00094-s001.zip › materials-4049643-supplementary.pdf]

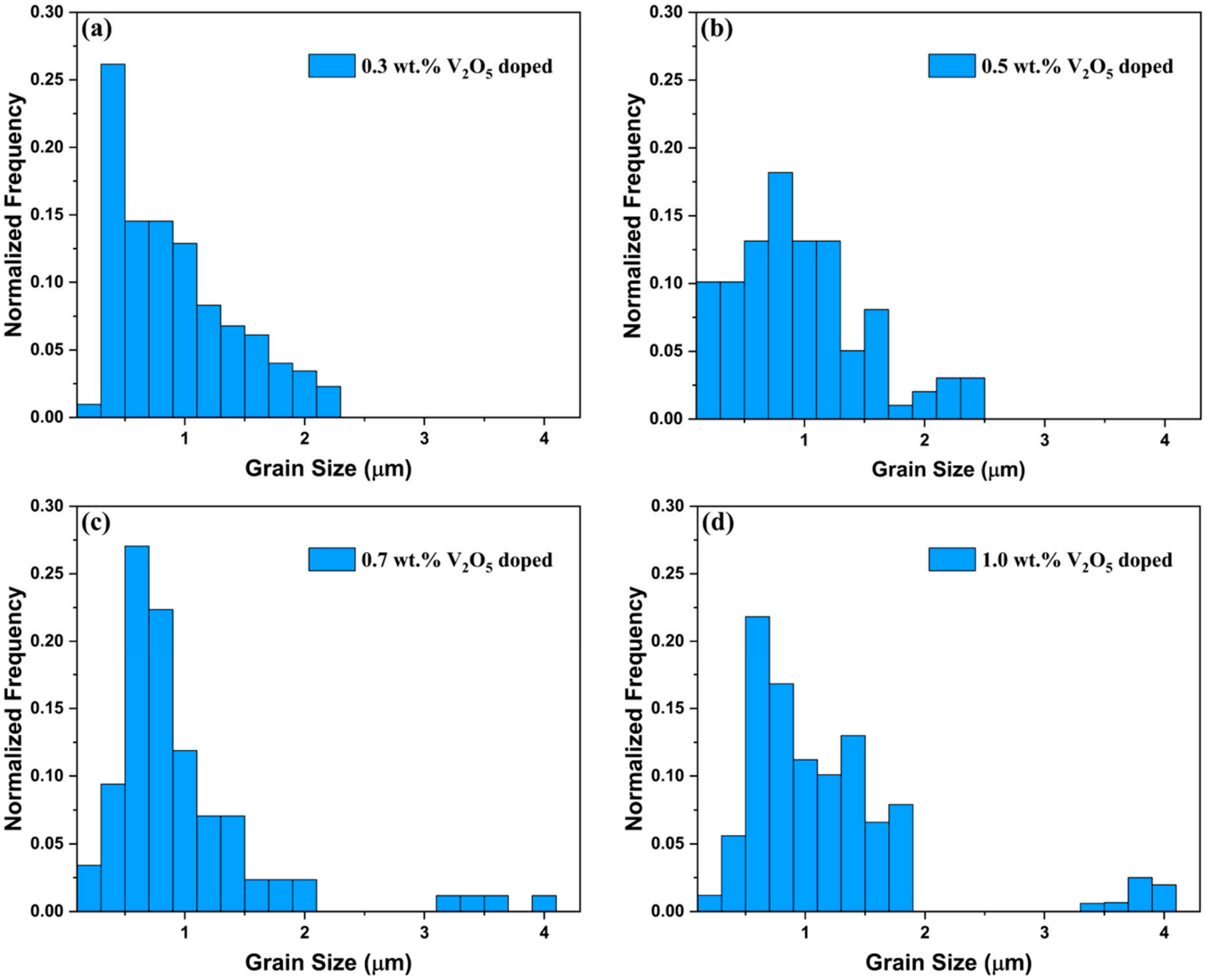

Supplementary Figure S1. Grain-size distribution histograms (probability density) for the 0.6LT–0.4LZT composite sintered with different V_2O_5 contents: (a) 0.3 wt.%, (b) 0.5 wt.%, (c) 0.7 wt.%, and (d) 1.0 wt.%. All histograms are plotted using a common grain-size axis to enable direct comparison. With increasing V_2O_5 content, the distributions progressively broaden and develop a pronounced high-size tail, while the central part of the distribution does not shift significantly. This behavior indicates the emergence of a minor population of abnormally coarsened LT grains rather than a uniform shift of the entire grain-size distribution.
